# [Retracted] miR‑200b and miR‑200c co‑contribute to the cisplatin sensitivity of ovarian cancer cells by targeting DNA methyltransferases 

**DOI:** 10.3892/ol.2025.14951

**Published:** 2025-03-04

**Authors:** Jue Liu, Xiaobo Zhang, Yuliang Huang, Qunfeng Zhang, Jianbin Zhou, Xiaodi Zhang, Xiaoxu Wang

Oncol Lett 17: 1453–1460, 2019; DOI: 10.3892/ol.2018.9745

Following the publication of this paper, it was drawn to the Editor's attention by a concerned reader that certain of the flow cytometric assay data shown in Fig. 2 on p. 1456 had already appeared in a pair of previously published articles written by different authors at different research institutes. Owing to the fact that the contentious data in the above article had already been published prior to its submission to *Oncology Letters*, the Editor has decided that this paper should be retracted from the Journal. The authors were asked for an explanation to account for these concerns, but the Editorial Office did not receive a reply. The Editor apologizes to the readership for any inconvenience caused.

